# Valproic Acid Treatment Inhibits Hypoxia-Inducible Factor 1α Accumulation and Protects against Burn-Induced Gut Barrier Dysfunction in a Rodent Model

**DOI:** 10.1371/journal.pone.0077523

**Published:** 2013-10-17

**Authors:** Hong-Min Luo, Ming-Hua Du, Zhi-Long Lin, Lin Zhang, Li Ma, Huan Wang, Wen Yu, Yi Lv, Jiang-Yang Lu, Yu-Li Pi, Sen Hu, Zhi-Yong Sheng

**Affiliations:** 1 Laboratory of Shock and Organ Dysfunction, Burns Institute, the First Hospital Affiliated to the People’s Liberation Army General Hospital, Beijing, China; 2 Obstetrics and Gynecology Department, the First Hospital Affiliated to the People’s Liberation Army General Hospital, Beijing, China; 3 Department of Pathology, the First Hospital Affiliated to the People’s Liberation Army General Hospital, Beijing, China; 4 Department of Ophtalmology, the First Hospital Affiliated to the People’s Liberation Army General Hospital, Beijing, China; Duke University Medical Center, United States of America

## Abstract

**Objective:**

Burn-induced gut dysfunction plays an important role in the development of sepsis and multiple organ dysfunction. Emerging evidence suggests that hypoxia-inducible factor-1α (HIF-1α) is critical in paracelluar barrier functions via regulating vascular endothelial growth factor (VEGF) and myosin light chain kinase (MLCK) expression. Previous studies have also demonstrated that histone deacetylase inhibitors (HDACIs) can repress HIF-1α. This study aims to examine whether valproic acid (VPA), a HDACI, protects against burn-induced gut barrier dysfunction via repressing HIF-1α-dependent upregulation of VEGF and MLCK expression.

**Methods:**

Rats were subjected to third degree 55% TBSA burns and treated with/ without VPA (300mg/kg). Intestinal barrier dysfunction was evaluated by permeability of intestinal mucosa to fluorescein isothiocyanate (FITC)-dextran and histologic evaluation. Histone acetylation, tight junction protein zonula occludens 1 (ZO-1), VEGF, MLCK and HIF-1α were measured. In addition, CaCO_2_ cells were transfected with siRNA directed against HIF-1α and were stimulated with CoCl2 (1mM) for 24 hours with/without VPA (2mM) followed by analysis of HIF-1α, MLCK, VEGF and ZO-1.

**Results:**

Burn insults resulted in a significant increase in intestinal permeability and mucosal damage, accompanied by a significant reduction in histone acetylation, ZO-1, upregulation of VEGF, MLCK expression, and an increase in HIF-1α accumulation. VPA significantly attenuated the increase in intestinal permeability, mucosa damage, histone deacetylation and changes in ZO-1 expression. VPA also attenuated the increased VEGF, MLCK and HIF-1α protein levels. VPA reduced HIF-1α, MLCK and VEGF production and prevented ZO-1 loss in CoCl2-stimulated Caco-2 cells. Moreover, transfection of siRNA directed against HIF-1α led to inhibition of MLCK and VEGF production, accompanied by upregulation of ZO-1.

**Conclusions:**

These results indicate that VPA can protect against burn-induced gut barrier dysfunction. These protective effects may be due to its inhibitory action on HIF-1α, leading to a reduction in intestinal VEGF and MLCK expression and minimizing ZO-1 degradation.

## Introduction

The development of systemic inflammatory response syndrome, sepsis and multiple organ dysfunction remain the common causes of morbidity and mortality in major burn injury, and it is generally accepted that the ischemic gut during shock phase may contribute to the development of sepsis and multiple organ dysfunction in burn patients [1-3]. Although there is a huge amount of bacteria and endotoxin distributed throughout the whole gut, gut-origin bacteremia and sepsis do not occur in a healthy individual because the intestinal epithelium functions as a barrier to prevent the escape of intraluminal bacteria and endotoxin to lymphatic system and blood. However, when an individual is insulted by a major burn, organ blood flow will be redistributed in favor of vital organs while blood flow to gut and other peripheral organs will be significantly decreased, resulting in gut barrier dysfunction and subsequent endotoxin and bacterial translocation, gut-origin sepsis and multiple organ dysfunction.

The integrity of gut barrier is mainly maintained by tight junctions of intestinal mucosa, which are composed of a large complex of proteins including the integral proteins such as claudins, occludin, and the peripheral membrane proteins such as zonula occludens 1 (ZO-1) [4,5]. ZO-1 is one of the most often investigated proteins and it is mainly responsible for connecting the intergal membrane proteins to the actin cytoskeleton and different types of signalling proteins [5]. Increasing evidence suggests that hypoxia-inducible factor-1 (HIF-1) plays critical roles in paracellular barrier functions, including intestinal epithelial barrier [6-11]. HIF-1 is an important transcription factor regulating the utilization of oxygen, nutrients and plays critical roles in phsysiological adaptations to hypoxia [12,13]. It is a heterodimer composed of an oxygen-inducible α subunit (HIF-1α) and an oxygen-independent subunit (HIF-1β) [14,15]. However, under normoxia conditions, HIF-1α is rapidly degraded due to hydroxylation of specific proline residues by prolyl hydroxylases (PHDs) [16]. Under hypoxia conditions, PHD activity is inhibited and HIF-1α begins to accumulate, and it is transported to the nucleus where it binds HIF-1β, forming the functional HIF-1 protein and regulating a number of target gene transcription [16]. MLCK and VEGF are two important downstream genes regulated by HIF-1, and previous studies have showed that they are potent modulators of cellular contacts [6,17-22]. Expression of MLCK and VEGF correlate with loss of ZO-1 and increased paracellular permeability [23-27]. Thus, efforts to attenuate the accumulation of HIF-1α may benefit burn patients who are at high risk of developing gut barrier dysfunction via the transcriptional repression of MLCK and VEGF expression. 

Valproic acid (VPA), a histone deacetylase inhibitor, has been shown to have protective effects on various hypoxia pathologies [28-32], and it has recently been found that SAHA, also a histone deacetylase inhibitor, significantly attenuated the accumulation of HIF-1α in macrophages cultured under hypoxia condition [33]. Furthermore, recent reports showed that blood-brain and blood-spinal cord barrier disruption were attenuated after VPA treatment [29,34]. However, the protective effects of VPA on burn-induced gut barrier dysfunction have not been confirmed. Thus, in this study, we aim to test the hypothesis that after major burn injury, VPA protects against the loss of ZO-1 through inhibiting the HIF-1α-dependent regulation of MLCK and VEGF expression, thereby attenuating the gut epithelial barrier dysfunction. Our present data demonstrate that VPA treatment significantly attenuates the burn-induced increase in intestinal permeability, mucosa damage, histone deacetylation and changes in ZO-1 expression. HIF-1α, VEGF and MLCK protein levels are also reduced after VPA treatment. In addition, the expression of VEGF and MLCK are upregulated in Caco-2 cells stimulated with CoCl2, and VPA treatment prevents these changes.

## Methods

### 1: Ethics statement

All animal experiments were approved by the Committee of Scientific Research of First Hospital Affiliated to General Hospital of PLA, China and were conducted in accordance with the National Institute of Health Guide for the Care and Use of Laboratory Animals.

### 2: Rat burn model and treatment

Male Sprague-Dawley rats (8-10 weeks, 240-260 g) were purchased from Experimental Animal Center of Military Medical Sciences of the Chinese PLA. The rats were housed in mesh cages in a room maintained at 25° C, illuminated with 12:12-h light-dark cycles, and provided with standard rodent chow and water ad libitum. The animals were randomly divided into four groups: sham scald with normal saline administration (sham+NS); sham scald with VPA administration (sham+VPA); scald with normal saline administration (scald+NS); scald with VPA administration (scald+VPA). Full-thickness burn injury occupying 55% of total body surface area was produced as described by Ikezu T et al [35]. Briefly, following a 12-h fast with water available ad libitum, rats were subjected to scald injury by immersing the back of the trunk for 15 s and the abdomen for 8 s in 80° C water under anesthesia with inhaled isoflurane (Yeeran Technology Limited, Beijing, China). Sham-burned rats were immersed into water at room temperature. Following burn injury, animals received a subcutaneous injection of 0.5 ml normal saline with 0.1 mg/kg of buprenorphine (Sigma, St. Louis, MO, USA) for pain control. The rats in sham+VPA group and scald+VPA group were subcutaneously given with VPA (300 mg/kg in 0.25 mL normal saline, Sigma, St. Louis, MO, USA) while the rats in sham+NS group and scald+NS group were subcutaneously injected vehicle (0.25 mL normal saline). Animals were recovered from anesthesia and returned to their cages with free acess to food and water. 

### 3: Tissue harvest

Animals were anesthetized and the blood were collected for intestinal permeability assay at 2 hours or 6 hours post-burn, then the animals were sacrificed for tissue harvest. Segments of distal small intestine were removed and snap frozen in liquid nitrogen before storage at -80° C for Western blot and ELISA assay or fixed in 4% paraformaldehyde for histologic evaluation and immunofluorescent staining.

### 4: Histologic evaluation

The paraformaldehyde-fixed intestines were embedded in paraffin, and cut in 2-μm sections. Hematoxylin and eosin staining of the intestine was performed by the Pathology Department of the First Hospital Affiliated to the People’s Liberation Army General Hospital. Then the sections were viewed under a light microscope and evaluated by two pathologists blinded to the experimental groups. The injury to the intestinal mucosa was scored using the grading system developed by Chiu et al. [36].

### 5: Intestinal permeability assay

An in vivo intestinal permeability assay was performed to assess gut epithelial barrier function as described by Schaper et al. [37]. Briefly, a midline laparotomy incision was performed 30 minutes before sacrificing the animals at the end of the experiment, and a 10-cm segment of distal ileum was isolated and ligated with 2-0 silk ties. Then, 1 ml 4 kDa FITC-dextran solution (10 mg/ml, diluted in phosphate-buffered saline, Sigma, St. Louis, MO, USA) was injected into the ligated lumen, after which the bowel was returned into the abdominal cavity and the abdomen was closed. Anesthesia was maintained for 30 minutes, and then blood was drawn from the abdominal aorta and centrifuged to separate the plasma. The plasma was stored at -80°C until quantification of fluorescence with a spectrophotometer (Synergy2, BioTek Multi-Detection Microplate reader, USA). The plasma FITC-dextran concentrations were calculated according to the standard curve.

### 6: Cell culture and treatments

Caco-2 (ATCC HTB-37, a gift from Department of Pharmacology, the First Hospital Affiliated to the People’s Liberation Army General Hospital, China) cell line was cultured in Dulbecco Modified Eagle medium supplemented with 10% fetal bovine serum, 1% nonessential amino acids, 2 mM L-glutamine, 100 U/mL penicillin, and 100 mg/mL streptomycin (all purchased from Life Technologies, Gaithersburg, MD, USA). Cells were seeded in 6-well flat-bottom plates until they reached 100% confluence. Then the cells were stimulated with or without CoCl2 (1 mM, Sigma, St. Louis, MO, USA)/VPA (2 mM). After 24 hours of stimulation, the culture supernatant was collected for determination of VEGF, and the cells were lysed for determination of HIF-1α, MLCK and ZO-1.

### 7: siRNA interference

Caco-2 cells were transfected (RNAiMAX, Life Technologies, Gaithersburg, MD, USA) with 50 nM siRNA targeting HIF-1α or HIF-1α scrambled control. siRNA duplexes were removed after 16 h, and Caco-2 cells were incubated for a further 8 h before CoCl2 stimulation.

### 8: Enzyme-Linked Immunosorbent Assay

The VEGF concentrations in gut tissue extrats of distal ileum and culture supernatant were measured using a commercial ELISA kit (R&D Systems, Minneapolis, MN, USA) according to the manufacturer’s protocol. The absorbance rate was read at 450 nm. The concentrations of the samples were calculated according to the standard curve. For the VEGF concentrations in gut tissues, the total protein concentration was determined by a protein assay kit (Applygen Technologies Inc, Beijing, China) and VEGF values were then normalized to protein concentration (in pg/mg of total protein).

### 9: Immunofluorescent staining

After deparaffinization, the intestine sections were rehydrated and incubated in citrate buffer (Zhongshan Jinqiao Biotechnology Co., Ltd., Beijing, China) for heat-induced antigen retrieval. After three washes with PBS, sections were incubated with 3% BSA (Zhongshan Jinqiao Biotechnology Co., Ltd., Beijing, China) for 30 minutes to block nonspecific binding sites. The sections were then incubated with primary antibodies against ZO-1 (1:100; Life Technologies, Gaithersburg, MD, USA) or acetyl histone H3K9 (1:100; Abcam, Cambridge, UK) at 4° C overnight. Then, sections were washed three times with PBS and incubated with Alexa Fluor 488 congugated anti-rabbit antibody (1:1000; Life Technologies, Gaithersburg, MD) in 1% BSA for 1 hour, followed by 3 washes with PBS and mounted using Antifade Solution (Applygen Technologies Inc, Beijing, China). Images were obtained using the Olympus fluorescence microscope (BX51-DP71) with exposure-matched settings.

### 10: Western blot analysis

The harvested gut tissues or cultured cells were placed in lysis buffer (50 mM Tris-HCl, PH 7.4; 150 mM NaCl; 1% NP-40; 0.1% SDS), then homogenized/lysed and centrifuged at 12,000 g for 10 minutes. Following centrifugation, the supernatant was collected and analyzed for protein concentration. Protein concentrations were determined using a protein assay kit (Applygen Technologies Inc, Beijing, China). Total protein (100 μg) was loaded onto a sodium dodecyl sulfate-polyacrylamide gel (SDS-PAGE gel) and run at 120 volts for 2 hours. After electrophoresis, the protein was transferred to a polyvinylidene difluoride membrane (PVDF; Applygen Technologies Inc, Beijing, China) and blocked for 2 hours in TBST (50 mM Tris; 150 mM NaCl; 0.05% Tween 20) containing 5% milk (Applygen Technologies Inc, Beijing, China). The membrane was then incubated with the primary antibodies against β-actin (1:1000; Zhongshan Jinqiao Biotechnology Co., Ltd., Beijing, China), acetyl histone H3K9 (1:400; Abcam, Cambridge, UK), ZO-1 (1:500; Life Technologies, Gaithersburg, MD, USA), MLCK (1:1000; Sigma, St. Louis, MO, USA), and HIF-1α (1:500; Novus Biologicals, Littleton, CO, USA) at 4° C overnight. After 3 washes in TBST, the membrane was then incubated with corresponding secondary antibody conjugated to horseradish peroxidase at room temperature for 30 minutes, and chemiluminescence detection was performed by using SuperECL Plus (Applygen Technologies Inc, Beijing, China). Films were developed using a standard photographic procedure. 

Quantitative analysis of detected bands was carried out by densitometer scanning (ImageJ). Briefly, the original image was converted to a 8-bit gray-scale image. Then, each lane was selected by a rectangle using the Rectangular Selections tool. After setting the rectangle in place on the last lane, a profile plot of each lane was drawn and each peak was enclosed by drawing a line across the base of the peak using the Straight Line selection tool. After that, the peak area of each lane was selected with the Wand tool and the area of each peak was measured. Finally, the area for each target protein in the Western blots were normalized to the β-actin loading control to get the relative intensities of each target protein in each lane.

### 11: Statistical analysis

SPSS 13.0 statistical software was used, and all results were expressed as mean ± SD. One-way ANOVA was used for comparison among all groups, followed by the Student-Newman-Keuls (SNK) test for comparison between two groups. Differences were considered to be statistically significant when *P* < 0.05.

## Results

### 1: VPA attenuates burn-induced intestinal injury

**Figure 1:**
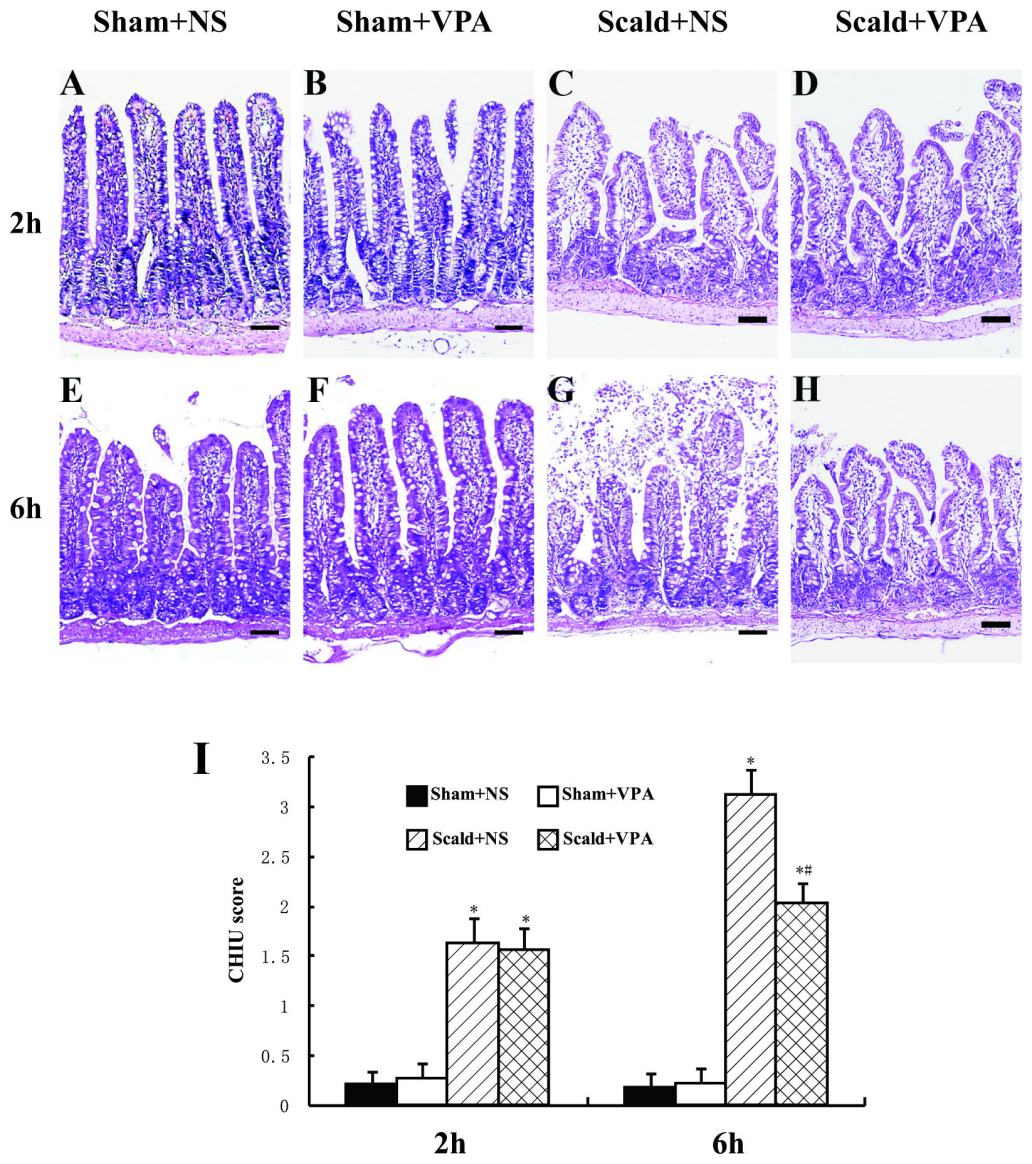
VPA attenuates burn-induced intestinal injury. Sections of distal ileum harvested 2 and 6 hours after 55% TBSA burn were stained with HE (A-H). A, E and B, F: representative image from sham+NS (A, E) and sham+VPA (B, F) group showing normal intestinal villi architecture; C and G: sections from scald+NS group at 2 and 6 hours, respectively; subepithelial space at villus tips coupled with moderate lifting (C) and massive lifting down sides of villi and some denuded tips (G) were observed; D and H: sections from scald+VPA group at 2 and 6 hours, respectively; intestinal villi architecture alterations were attenuated compared to control animals at 6 hours, without massive lifting down sides of villi. Black bar = 100 μm. I: intestinal injury was scored using the Chiu’s grading system. Chiu’s scores were significantly elevated both at 2 and 6 hours post-burn; VPA treatment significantly attenuated intestinal injury at 6 hours post-burn. Data were expressed as mean values ± SD (n=5). *P<0.05, compared with Sham+NS group; # *P* < 0.05, compared with Scald+NS group.

Histologic evaluation of burn-induced injury to the intestinal mucosa was performed based on the Chiu’s grading system [36]. The histopathologic analysis of the sham burned animals (sham+NS and sham+VPA) showed a normal mucosal pattern. The villi were packed, tall, and intact (Figure 1A, E). Compared with the sham burned animals, burn insults caused a significant mucosal damage. There was subepithelial space at villus tips coupled with moderate lifting at 2 hours post-burn (Figure 1C), and massive lifting down sides of villi and some denuded tips were observed at 6 hours post-burn (Figure 1G). However, VPA treatment significantly attenuated the mucosal damage at 6 hours post-burn (*P* < 0.05, Figure 1). No significant difference was observed between scald+NS group and scald+VPA group at 2 hours post-burn (*P* > 0.05, Figure 1).

### 2: VPA prevents burn-induced increase in intestinal permeability

**Figure 2:**
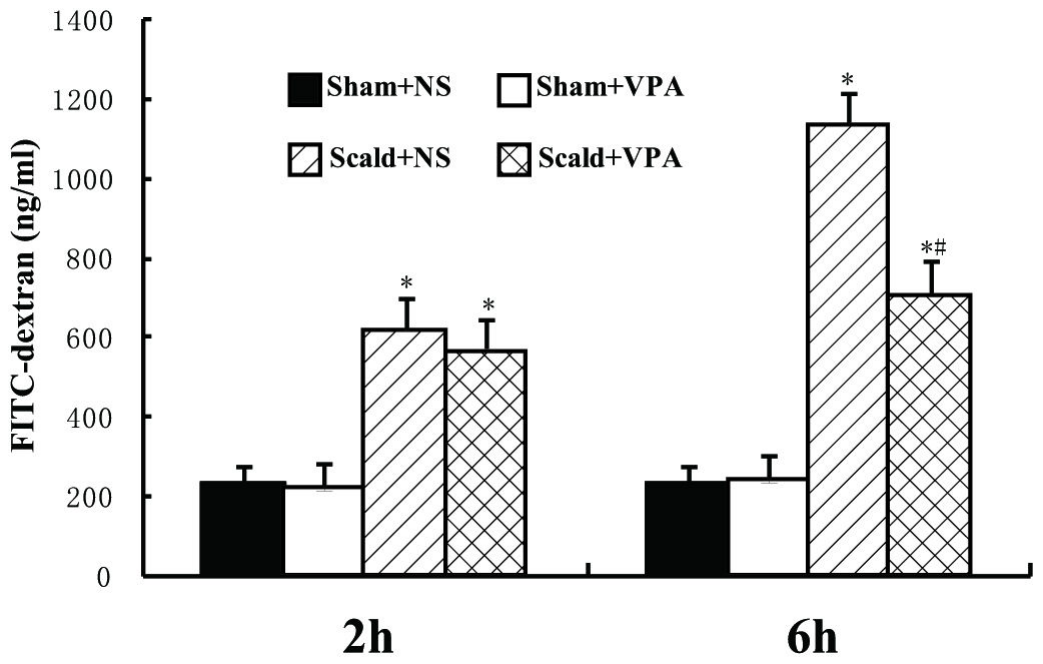
VPA prevents burn-induced increase in intestinal permeability. Intestinal permeability was assessed by measuring FITC-dextran in the systemic circulation after intraluminal injection of 4 kDa FITC-dextran. The intestinal permeability was significantly increased at 2 hours and 6 hours post-burn. VPA treatment significantly inhibited the increase in intestinal permeability at 6 hours post-burn. Data were expressed as mean values ± SD (n=5). * *P* < 0.05, compared with Sham+NS group; # *P* < 0.05, compared with Scald+NS group.

The intestinal permeability was evaluated in an in vivo assay using FITC-Dextran. The intestinal permeability was significantly increased at 2 hours post-burn, and it was increased about 6 fold within 6 hours post-burn (all *P* < 0.05, Figure 2). VPA treatment significantly inhibited the increase in intestinal permeability at 6 hours post-burn (*P* < 0.05, Figure 2). However, there was no significant difference in permeability between scald+NS group and scald+VPA group at 2 hours post-burn (*P* > 0.05, Figure 2). 

### 3: VPA increases acetylation of histone H3 at K9

**Figure 3:**
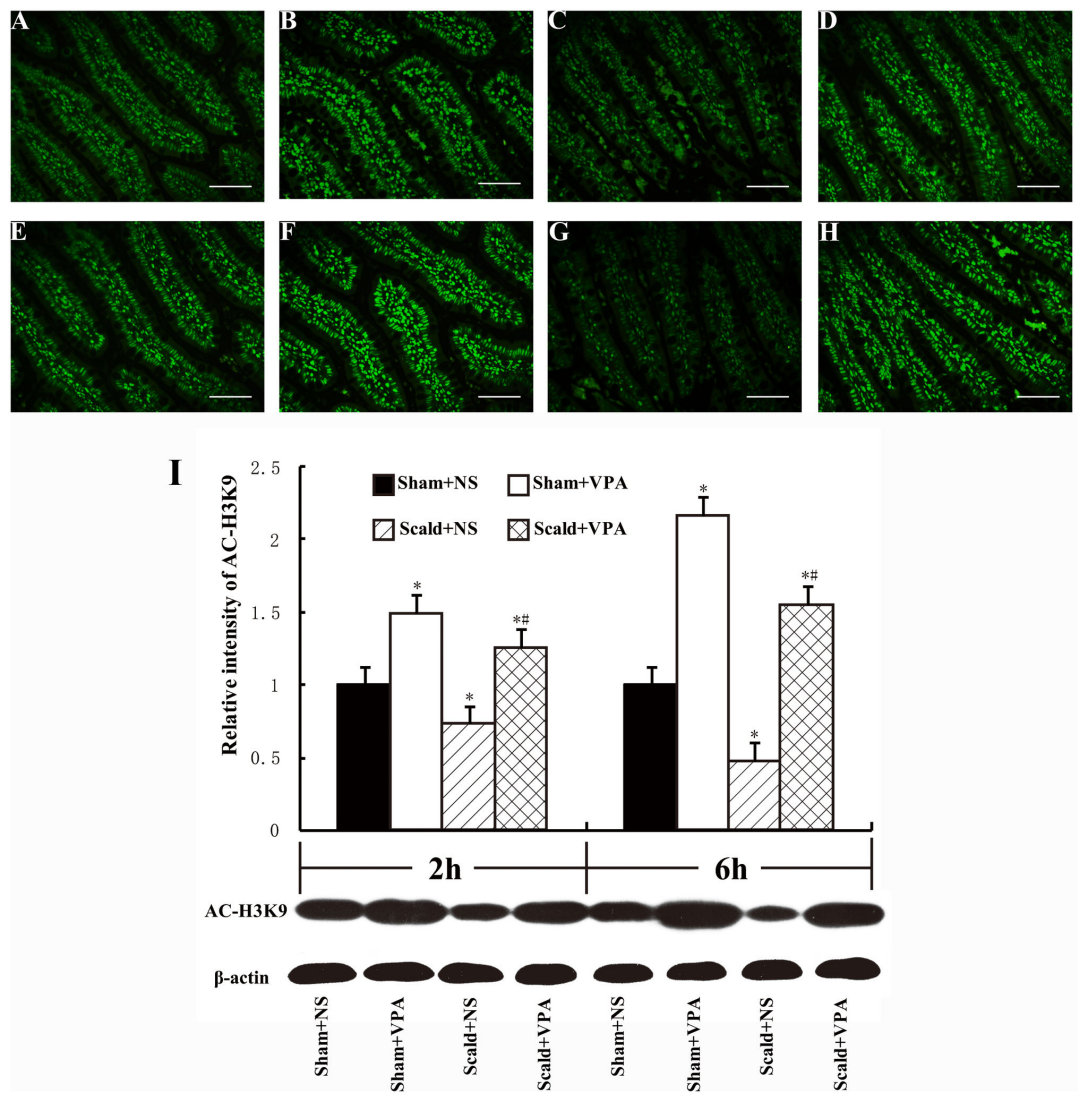
VPA increases acetylation of histone H3 at K9. Sections of distal ileum harvested 2 and 6 hours after 55% TBSA burn were labeled for Ac-H3K9 (A-H). A and E: representative image from sham+NS group showing that histone acetylation ubiquitously existed in normal intestinal cells; C and G: sections from scald+NS group at 2 and 6 hours, respectively; fluorescence intensity of Ac-H3K9 was decreased after burn injury compared to sham-burned rats; B, F and D, H: sections from sham+VPA (B, F) and scald+VPA (D, H), respectively; VPA treatment increased the fluorescence intensity of Ac-H3K9 both at sham-burned and burned rats. White bar = 100 μm. I: Western Blot analysis of intestinal Ac-H3K9. Burn insults resulted in a significant reduction in intestinal Ac-H3K9 levels and VPA treatment significantly increased the levels of intestinal Ac-H3K9 at 2 and 6 hours post-burn. Protein bands quantified by densitometry were expressed as mean values ± SD (n=5). * *P* < 0.05, compared with Sham+NS group. # *P* < 0.05, compared with Scald+NS group.

To assess the effects of VPA on acetylation of histone protein, acetylation of H3K9, a reliable marker for acetylation of histone protein [32], was analyzed by Western blot and immunoflourescence staining with anti-acetyl histone H3K9 (Ac-H3K9) antibody. The immunoflourescence staining of Ac-H3K9 showed an ubiquitous existence of histone acetylation in normal intestinal cells (Figure 3A, E). However, the intestinal histone was less acetylated at 2 and 6 hours post-burn (Figure 3C, G), and VPA treatment markedly increased the acetylation of histone both in normal or burned rats (Figure 3B, D, F and H). Western blot analysis of Ac-H3K9 confirmed our immunoflourescence staining results that VPA treatment significantly increased the levels of intestinal Ac-H3K9 at 2 and 6 hours post-burn (Figure 3I, all *P* < 0.05).

### 4: VPA prevents loss of ZO-1

**Figure 4:**
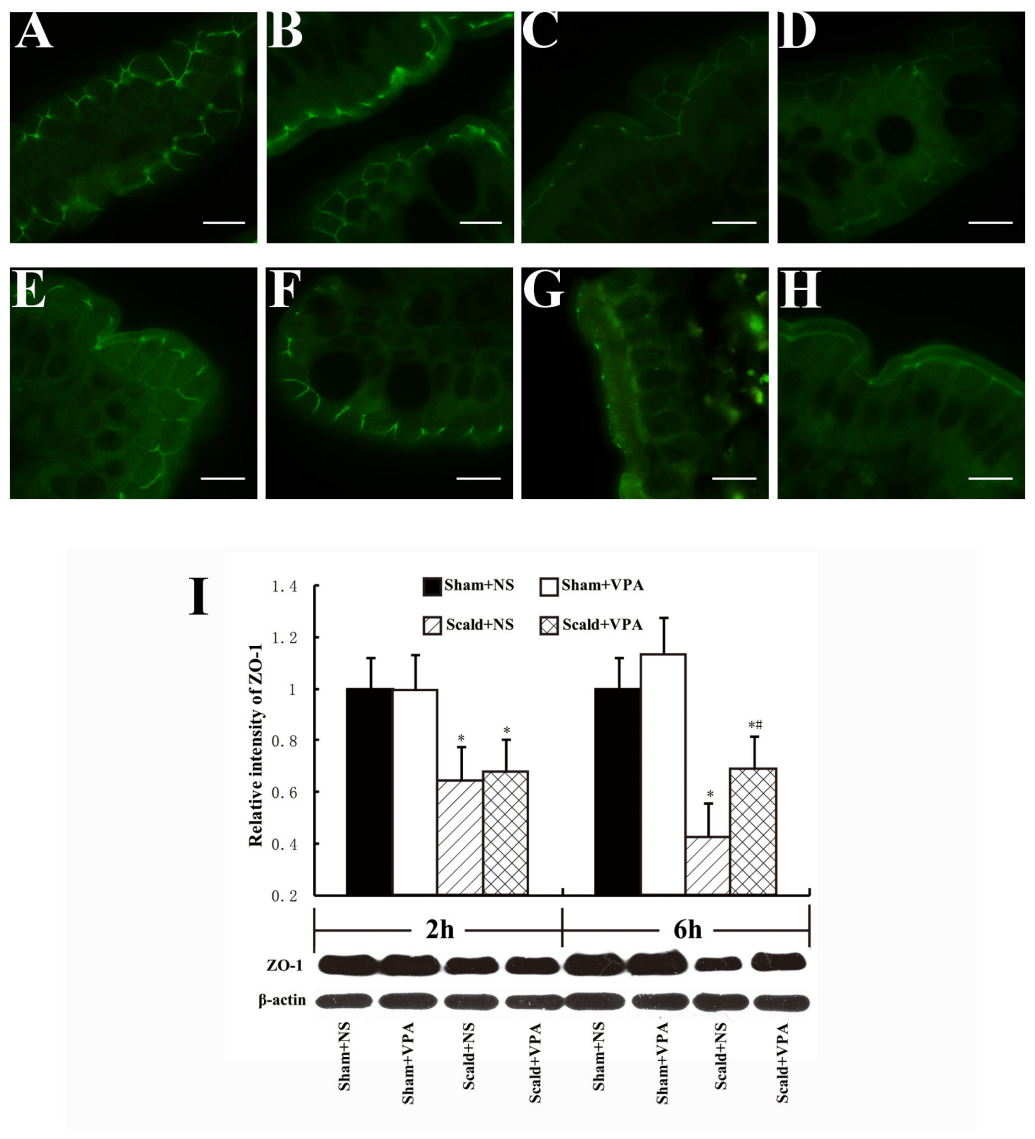
VPA prevents loss of ZO-1. Sections of distal ileum harvested 2 and 6 hours after 55% TBSA burn were labeled for ZO-1 (A-H). A, E and B, F: representative image from sham+NS (A, E) and sham+VPA (B, F) group showing normal intestinal ZO-1 expression; ZO-1 was densely and continuously distributed along the the apical membrane of the epithelial cells; C and G: sections from scald+NS group at 2 and 6 hours, respectively; burn insults caused loss of ZO-1 expression at 2 hours post-burn (C), and the burn-induced loss of ZO-1 was more pronounced at 6 hours post-burn , resulting in a disruption in zo-1 continuity (G); D and H: sections from scald+VPA group at 2 and 6 hours, respectively; the zo-1 continuity were improved compared to control animals at 6 hours. White bar = 10 μm. I: Western Blot analysis of intestinal ZO-1. Burn insults resulted in a significant reduction in intestinal ZO-1 levels at 2 and 6 hours, and VPA treatment significantly increased the intestinal ZO-1 expression at 6 hours post-burn. Protein bands quantified by densitometry were expressed as mean values ± SD (n=5). * *P* < 0.05, compared with Sham+NS group. # *P* < 0.05, compared with Scald+NS group.

To assess the effects of VPA on the expression of ZO-1, a member of tight junction proteins, immunoflourescence and Western blot analysis were performed. In sham-burned animals, ZO-1 was densely and continuously distributed along the apical membrane of the epithelial cells (Figure 4A, E). Burn insults caused loss of ZO-1 expression at 2 hours post-burn (Figure 4C), and the burn-induced loss of ZO-1 was more pronounced at 6 hours post-burn, resulting in a disruption of ZO-1 continuity (Figure 4G). After the treatment with VPA, the loss of ZO-1 was attenuated, and the ZO-1 continuity was improved at 6 hours post-burn (Figure 4H). The expression pattern of ZO-1 was similar between scald+NS group and scald+VPA group at 2 hours post-burn (Figure 4C, D).

Western blot analysis of ZO-1 confirmed our findings as shown by immunoflourescence staining. Burn insults resulted in a significant reduction in intestinal ZO-1 expression and VPA treatment attenuated degradation of ZO-1 at 6 hours post-burn (all *P* < 0.05, Figure 4I).

### 5: VPA reduces burn-induced increase in VEGF and MLCK

**Figure 5:**
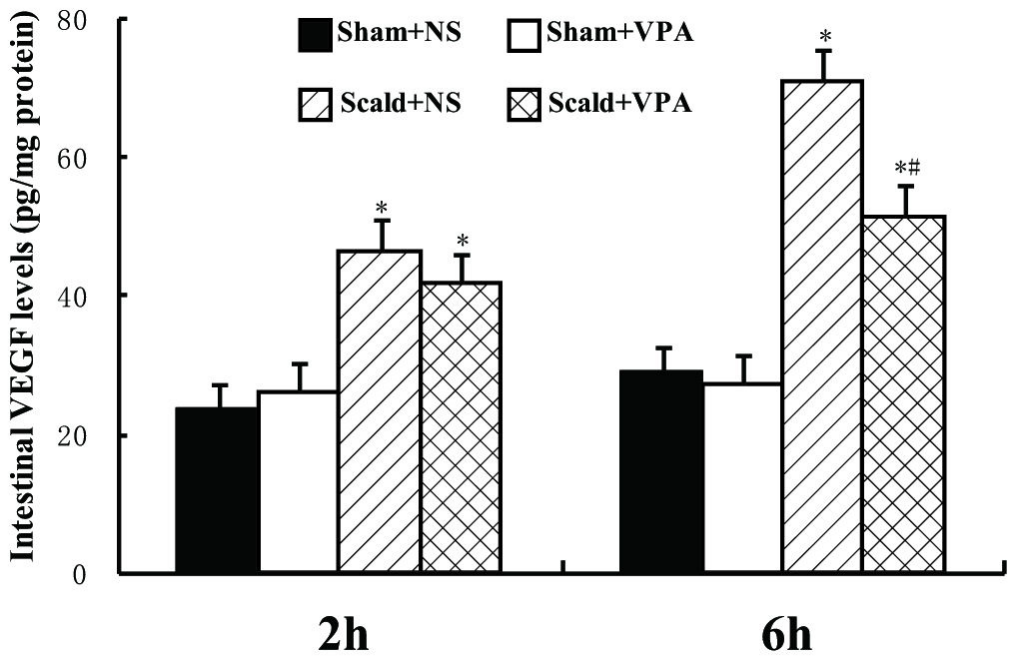
VPA reduces burn-induced increase in VEGF. Intestinal levels of VEGF were determined by ELISA. The intestinal VEGF was significantly increased at 2 hours and 6 hours post-burn. VPA treatment significantly decreased intestinal VEGF at 6 hours post-burn. Data were expressed as mean values ± SD (n=5). * *P* < 0.05, compared with Sham+NS group; # *P* < 0.05, compared with Scald+NS group.

**Figure 6:**
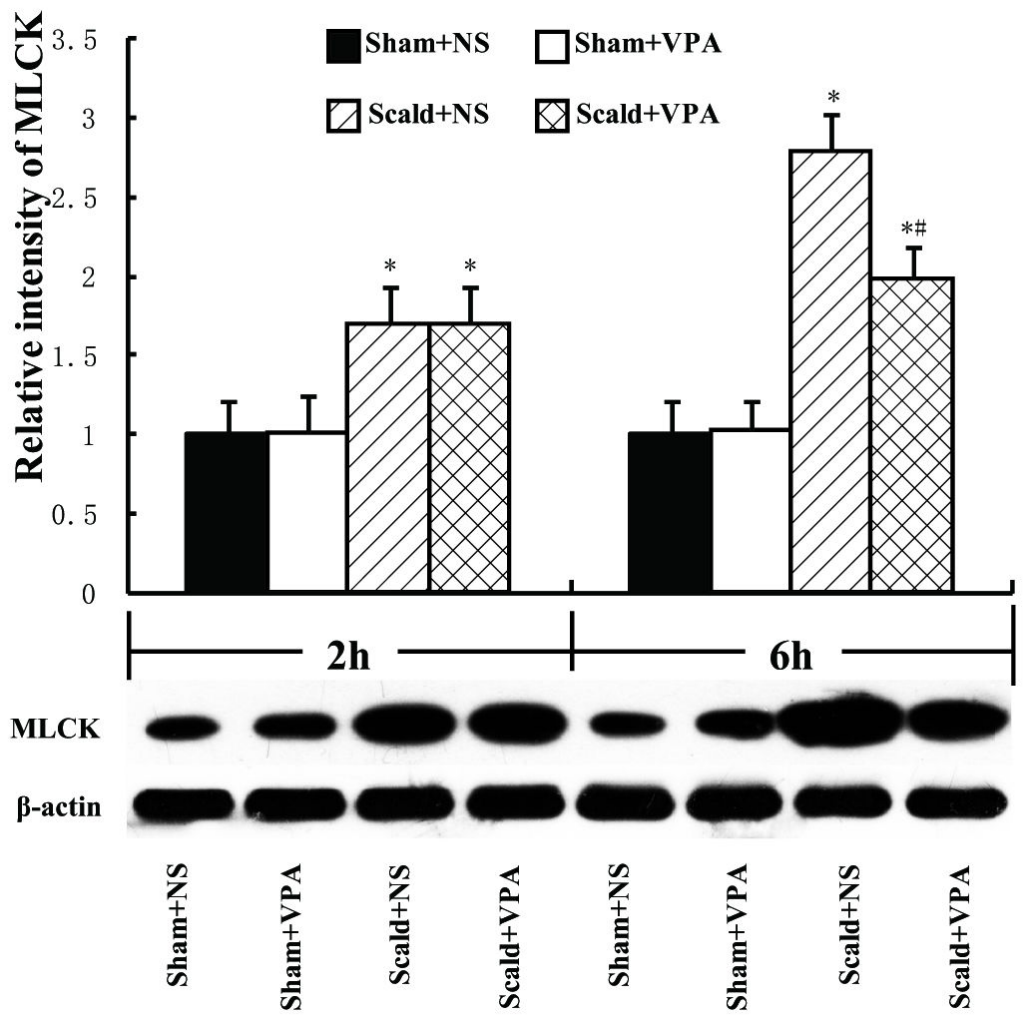
VPA reduces burn-induced increase in MLCK. Intestinal levels of MLCK were determined by Western Blot analysis. The intestinal MLCK was significantly increased at 2 hours and 6 hours post-burn. VPA treatment significantly decreased intestinal MLCK at 6 hours post-burn. Protein bands quantified by densitometry were expressed as mean values ± SD (n=5). * *P* < 0.05, compared with Sham+NS group; # *P* < 0.05, compared with Scald+NS group.

Since VEGF and MLCK proteins are two critical proteins involved in burn-induced gut epithelial barrier dysfunction, we performed the ELISA and Western blot assay to determine VEGF and MLCK levels in the distal ileum respectively. Burn insults resulted in a moderate increase in levels of intestinal VEGF at 2 hours post-burn, and a more pronounced increase at 6 hours post-burn (all *P* < 0.05, Figure 5). VPA treatment significantly reduced the intestinal levels of VEGF at 6 hours post-burn compared with scald+NS group (*P* < 0.05, Figure 5). There was no significant difference in levels of VEGF between scald+NS group and scald+VPA group at 2 hours post-burn (*P* > 0.05, Figure 5). The expression pattern of intestinal MLCK was similar to that of VEGF. Burn insults caused a significant increase in MLCK levels compared with sham-burned animals (*P* < 0.05, Figure 6), and VPA treatment significantly attenuated this increase at 6 hours post-burn (*P* < 0.05, Figure 6), but not at 2 hours post-burn (*P* > 0.05, Figure 6).

### 6: VPA inhibits burn-induced accumulation of HIF-1α

**Figure 7:**
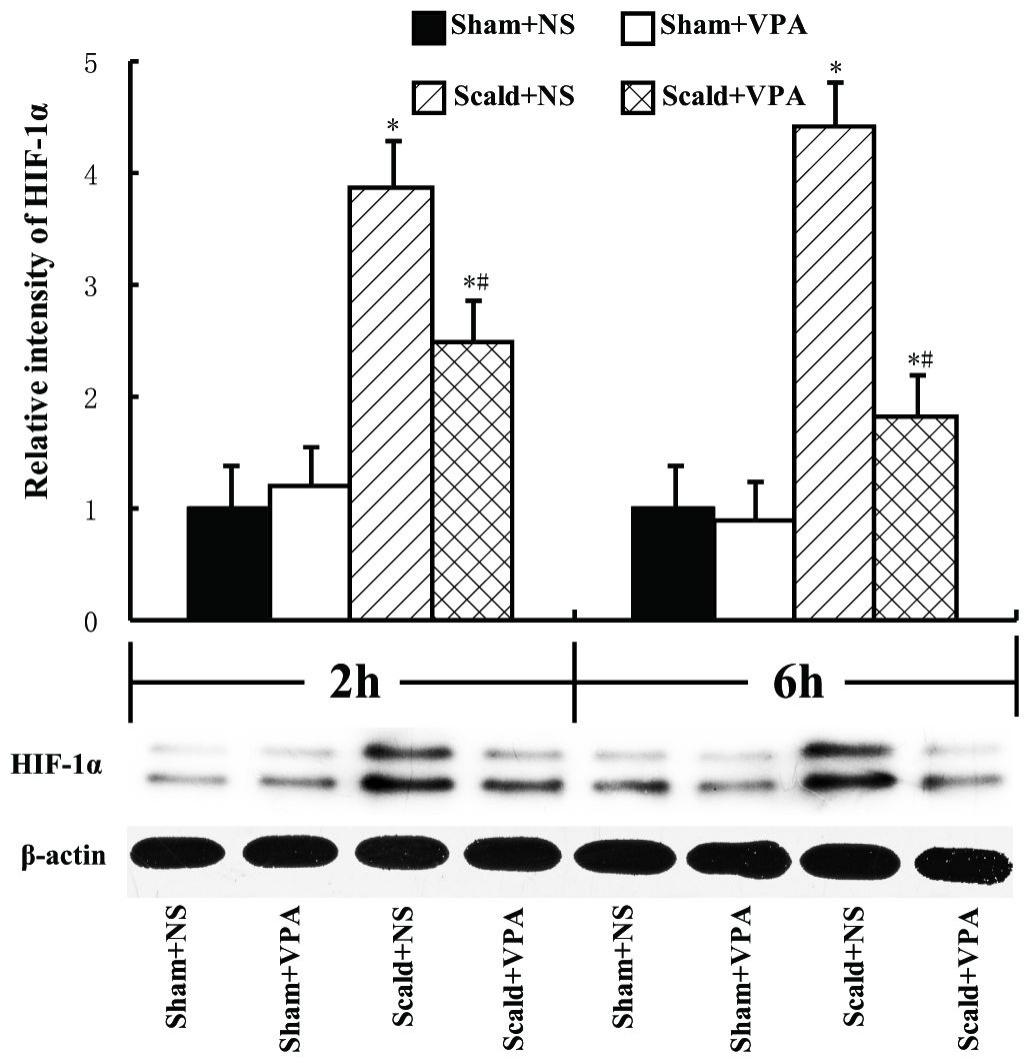
VPA inhibits burn-induced accumulation of HIF-1α. Intestinal levels of HIF-1α were determined by Western Blot analysis. The intestinal HIF-1α was significantly increased at 2 hours and 6 hours post-burn. VPA treatment significantly decreased intestinal HIF-1α at 2 and 6 hours post-burn. Protein bands quantified by densitometry were expressed as mean values ± SD (n=5). * *P* < 0.05, compared with Sham+NS group; # *P* < 0.05, compared with Scald+NS group.

Since HIF-1 is an important transcription factor for multiple genes including VEGF and MLCK, the intestinal levels of HIF-1α were measured by Western blot analysis. Burn insults significantly increased the levels of HIF-1α both at 2 and 6 hours post-burn , and VPA treatment markedly inhibited the accumulation of HIF-1α both at 2 and 6 hours post-burn (all *P* < 0.05, Figure 7).

### 7: VPA prevents ZO-1 loss via repressing CoCl2-induced stabilization of HIF-1α and production of VEGF and MLCK in Caco-2 cells

**Figure 8:**
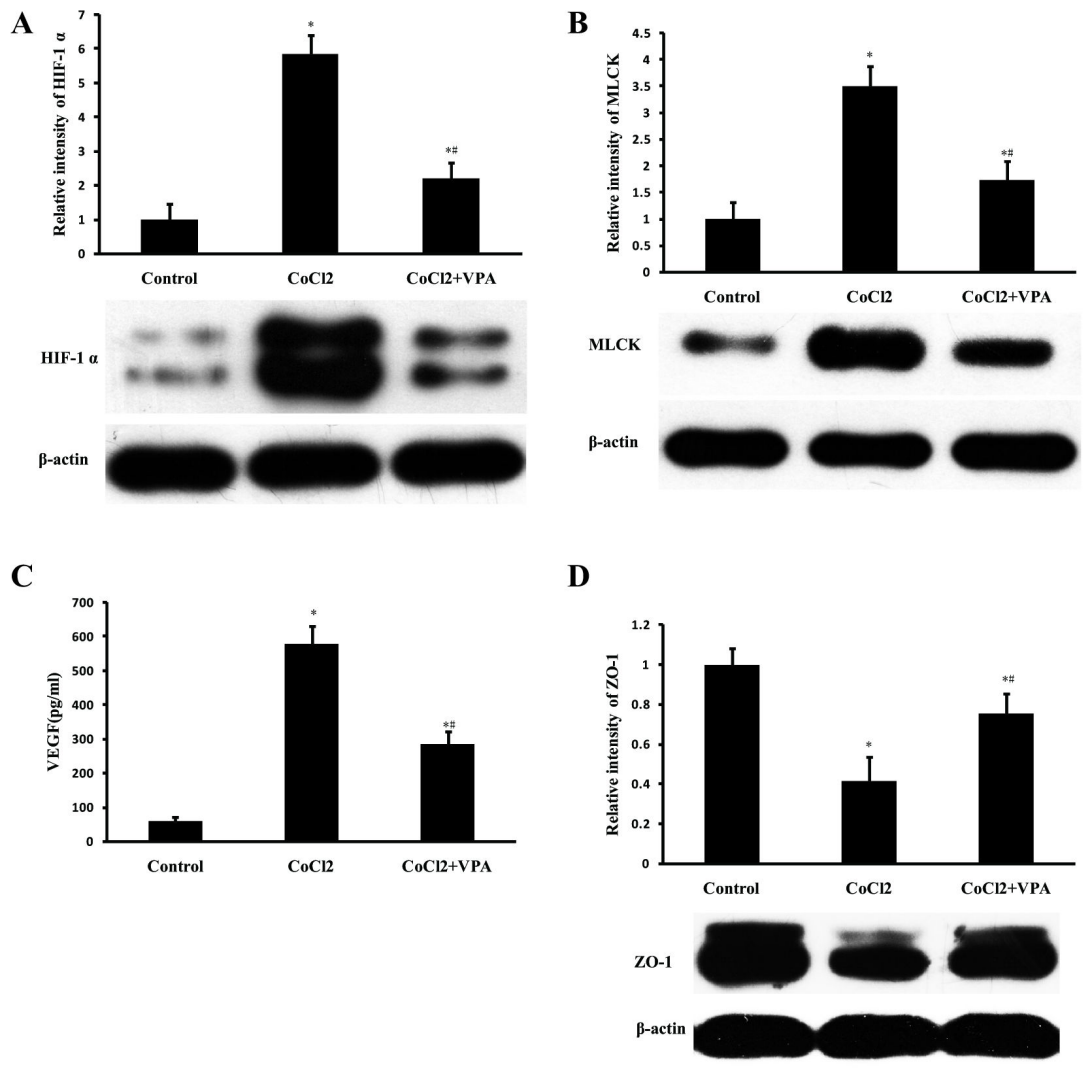
VPA reduces HIF-1α, MLCK and VEGF production and prevents ZO-1 loss in CoCl2-stimulated Caco-2 cells. Caco-2 cells were stimulated with or without CoCl2 (1 mM)/VPA (2 mM). After 24 hours of stimulation, the culture supernatant was collected for determination of VEGF by ELISA and the cells were lysed for determination of HIF-1α, MLCK and ZO-1 by Western Blot analysis. VPA treatment significantly repressed the CoCl-induced stabilization of HIF-1α (A), upregulation of MLCK (B), VEGF (C) and reduction of ZO-1 (D). Data were expressed as mean values ± SD (n=3), * *P* < 0.05, compared with control group; # *P* < 0.05, compared with CoCl2 group.

**Figure 9:**
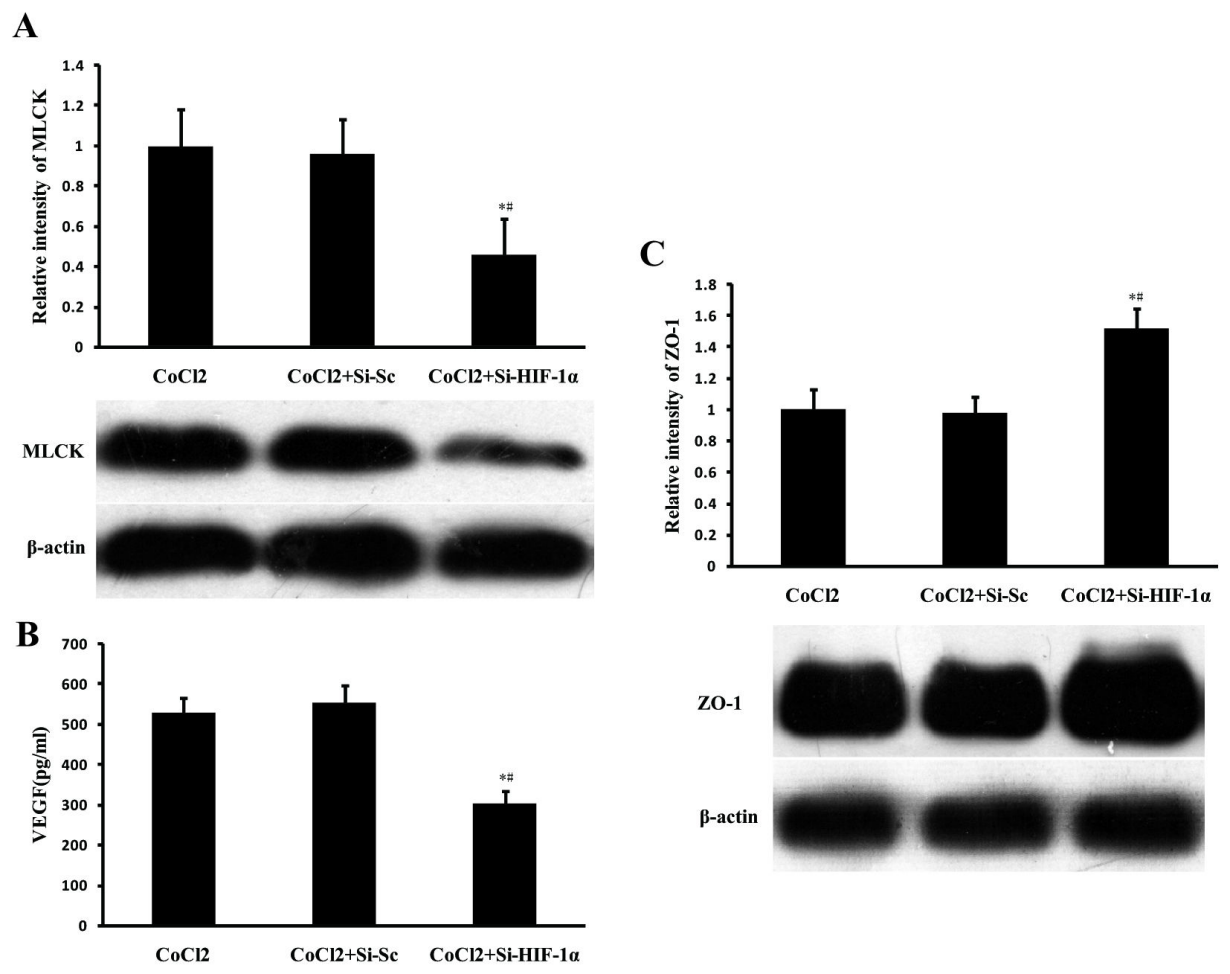
Upregulation of VEGF, MLCK and reduction in ZO-1 after CoCl2 stimulation is mediated partially through HIF-1α. Caco-2 cells were transfected with 50 nM siRNA targeting HIF-1α or HIF-1α scrambled control. siRNA duplexes were removed after 16 h, and Caco-2 cells were incubated for a further 8 h before CoCl2 stimulation. After 24 hours of CoCl2 stimulation, the culture supernatant was collected for determination of VEGF by ELISA and the cells were lysed for determination of HIF-1α, MLCK and ZO-1 by Western Blot analysis. Expression of MLCK (A) and VEGF (B) were significantly reduced after HIF-1α siRNA transfection, accompanied by upregulation of ZO-1 (C). Data were expressed as mean values ± SD (n=3), * *P* < 0.05, compared with CoCl2 group; # *P* < 0.05, compared with CoCl2+Si-Sc group.

The above findings show that HIF-1α, VEGF, and MLCK are increased after major burn injury, however, the mechanism responsible for the upregulation of VEGF and MLCK is not well understood. MLCK and VEGF are two important downstream genes regulated by HIF-1, and previous studies have showed that they are potent modulators of cellular contacts [6,17-22]. To examine this hypothesis, Caco-2 cells were stimulated with CoCl2 and treated with VPA for 24 hours. CoCl2 stimulation resulted in a significant increase in accumulation of HIF-1α (*P* < 0.05, Figure 8A), accompanied by upregulation of MLCK (*P* < 0.05, Figure 8B), VEGF (*P* < 0.05, Figure 8C) and reduction in ZO-1 levels (*P* < 0.05, Figure 8D). VPA treatment significantly repressed the CoCl-induced stabilization of HIF-1α, upregulation of MLCK, VEGF and reduction in ZO-1 (all *P* < 0.05, Figure 8). To examine whether the upregulation of both MLCK and VEGF were HIF-1α mediated, loss-of-function approach was used to knockdown HIF-1α expression by siRNA directed against HIF-1α. Caco-2 cells were transfected with siRNA targeting HIF-1α and stimulated with CoCl2. Expression of MLCK and VEGF were significantly reduced after HIF-1α siRNA transfection, accompanied by upregulation of ZO-1 (all *P* < 0.05, Figure 9).

## Discussion

This study demonstrates that VPA treatment has protective effects on burn-induced gut epithelial barrier dysfunction. Our data support the hypothesis that major burn injury induces HIF-1α accumulation, which activates VEGF and MLCK gene transcription, leading to loss and redistribution of ZO-1 and the subsequent increase in gut epithelial barrier permeability. Our results further demonstrate that VPA treatment inhibits HIF-1α accumulation, resulting in reduced VEGF and MLCK levels, which in turn attenuate ZO-1 degradation and redistribution, and thus protects against burn-induced gut epithelial barrier dysfunction.

The gastrointestinal tract is known to be a large pool of bacteria and endotoxin, and the intestinal epithelial barrier appears to play a critical role in preventing the translocation of luminal bacteria and endotoxin into systemic organs and tissues via lymphatic channel and blood stream. It has also been recognized that intestinal epithelial barrier dysfunction and increased intestinal permeability after thermal injury and may be the inciting incident that ultimately leads to SIRS and MODS [1-3]. In this study, we used a rat model of 55% TBSA scald injury in order to induce a significant changes in gut epithelial barrier function. In consistent with other studies [24,38,39], we noted that burn insults resulted in a significant mucosal damage and increased intestinal permeability both at 2 and 6 hours post-injury. 

It has been previously demonstrated that hemorrhagic shock is associated with acetylation imbalance of cardiac, lung, and liver histones, and that it can be restored by HDAC inhibitors, including VPA [40,41]. In this study, we found that the levels of intestinal Ac-H3K9 were reduced and administration of VPA significantly elevated Ac-H3K9 levels. This indicates that intestinal acetylation balance is disturbed after burn injury and VPA treatment can restore acetylation homeostasis. Furthermore, it has recently been shown that VPA treatment significantly attenuates ischemia-induced blood-brain barrier [34] and blood-spinal cord barrier disruption [29]. However, the effects of VPA on burned-induced gut epithelial barrier disruption has not been studied. Thus, we evaluated the effects of VPA on burned-induced gut epithelial barrier disruption using the Chiu’s grading system and intestinal permeability assay. In VPA-treated animals, attenuation of burn-induced mucosal damage and intestinal permeability were only observed at 6 hours, but not at 2 hours post-burn. Since ZO-1 is one of the tight junction proteins, which maintain the integrity of gut epithelial barrier, and recent studies have shown that burn insults cause changes in the ZO-1 expression [18,24,37], therefore, we decided to investigate the effects of VPA on expression of intestinal ZO-1. We found that burn insults resulted in a significant reduction in intestinal ZO-1, which is in consistent with other studies, and that VPA treatment attenuated alterations of ZO-1 at 6 hours, but not at 2 hours post-burn. This indicates that the protective effects of VPA on gut epithelial barrier dysfunction may be mediated through regulation of gene transcription and protein expression, processes which take time to cause a measurable effect.

Upregulation of VEGF and MLCK expression have been implicated in paracellular barrier dysfunction by changing the expression of the tight junction proteins in various models [17,19,24,42-44], and previous studies showed that VPA repressed expression of VEGF [45,46]. Thus, we further evaluated the effects of VPA on expression of intestinal VEGF and MLCK following burn injury. Our findings confirmed that intestinal VEGF and MLCK expression were up-regulated both at 2 and 6 hours post-burn. Furthermore, VPA treatment significantly reduced the expression of VEGF and MLCK at 6 hours post-burn.

VEGF and MLCK are both downstream genes regulated by hypoxia-inducible factor-1 (HIF-1), and increasing evidence suggests that HIF-1 is a key regulator in paracelluar barrier function via regulating VEGF and MLCK expression [6,7]. A recent study has shown that SAHA, also a histone deacetylase inhibitor, significantly attenuated the accumulation of HIF-1α in macrophages cultured under hypoxic condition [33]. In the present study, we found that intestinal HIF-1α was significantly increased both at 2 and 6 hours post-burn, and VPA treatment attenuated the accumulation of HIF-1α at 2 and 6 hours post-burn. The effects of VPA on HIF-1α occurred earlier than that on VEGF and MLCK expression, indicating that the protective effects of VPA on burn-induced intestinal epithelial barrier disruption may be mediated through its inhibitory effects on HIF-1α. In order to confirm that the protective effects of VPA on gut epithelial barrier were mediated through repression of HIF-1α, we used CoCl2, a PHD inhibitor, to induce HIF-1α accumulation. We found that the protein levels of HIF-1α, MLCK and VEGF were significantly elevated while that of ZO-1 were decreased, and these changes could be repressed by VPA treatment. Furthermore, expression of MLCK and VEGF were significantly downregulated after HIF-1α siRNA transfection, accompanied by upregulation of ZO-1. These results indicate that VPA prevents gut epithelial barrier dysfunction by repressing HIF-1α.

It has been documented that HDACIs, including VPA, were able to repress HIF-1 function, however, the mechanism is not fully elucinated. Histone deacetylases compose a group of enzymes that can remove the acetyl groups from N-ε-lysines of histones as well as many other non-histone proteins [47,48]. HIF-1α is easily detectable from the immunoprecipitates by using anti-acetyl-lysine antibodies and more recently, several studies showed direct detection of HIF-1α in immunoblotting with anti-acetyl-lysine antibodies [48]. It is proposed that acetylation of HIF-1α may induce degration of HIF-1α through promoting HIF-1α recognition and ubiquitination by VHL [48]. Therefore, one possibility is that HDACIs decrease the stability of HIF-1α through enhancing the acetylation of HIF-1α and/or other proteins involved in modulating the degradation of HIF-1α, thus accelerating the degradation of HIF-1α [48]. Further study is needed to identify the targets and define the specific acetylation sites responsible for HDACI-mediated HIF-1 repression. 

However, it should be noted that HIF-1 is a transcription factor which regulates a large number of hypoxia-induced genes other than VEGF and MLCK; and others have reported that HIF-1 and its downstream genes such as the multidrug resistance (MDR1) gene [49], intestinal trefoil factor [50], CD73 [50], adenosine A2B receptor [51] and mucin-3 [52] were implicated in the maintenance of barrier function during hypoxia. Different results between those mentioned above and ours may be due to the differences in models and the time points chosen to evaluate the barrier function. For example, they mainly focused on the role of HIF-1 in chronic hypoxia environment such as tumors and TNBS colitis, in which hypoxia develops slowly and the individual can adapt to hypoxia in a milder way. However, in our burn model, intestinal hypoxia develops quickly, and the rapid accumulation of HIF-1 resulted in a significant upregulation of VEGF and MLCK, which led to loss of ZO-1 and barrier dysfunction. Therefore, the role of HIF-1 in barrier function may be complicated, and drugs or other approaches targeting HIF-1 should be temporal specific.

In summary, VPA treatment protects against burn-induced gut epithelial barrier dysfunction by attenuating accumulation of HIF-1α, leading to a reduction in intestinal VEGF and MLCK expression and minimizing ZO-1 degradation.
